# Mast cell tryptase enhances wound healing by promoting migration in human bronchial epithelial cells

**DOI:** 10.1080/19336918.2021.1950594

**Published:** 2021-07-24

**Authors:** Sofia Mogren, Frida Berlin, Sangeetha Ramu, Asger Sverrild, Celeste Porsbjerg, Lena Uller, Cecilia K Andersson

**Affiliations:** a Department of Experimental Medical Science, Lund University, Lund, Sweden; b Department of Respiratory Medicine, Bispebjerg and Frederiksberg Hospital, Copenhagen, Denmark

**Keywords:** Mast cell, tryptase, epithelial cells, wound healing, protease activated receptor 2

## Abstract

Epithelial damage and increase of intraepithelial mast cells (MC) are characteristics of asthma. The role of MC mediator tryptase and the protease-activated receptor-2 (PAR2) on epithelial wound healing is not fully investigated. Stimulation of bronchial epithelial cells (BECs) with tryptase promoted gap closure, migration and cellular speed compared to controls. Stimulated BECs had higher expression of migration marker CD151 compared to controls. Proliferation marker KI67 was upregulated in tryptase-stimulated BECs compared to controls. Treatment with PAR2 antagonist I-191 reduced gap closure, migration and cell speed compared to BECs stimulated with tryptase. We found that tryptase enhances epithelial wound healing by increased migration and proliferation, which is in part regulated via PAR2. Our data suggest that tryptase might be beneficial in tissue repair under baseline conditions. However, in a pathological context such as asthma with increased numbers of activated MCs, it might lead to epithelial remodeling and loss of function.

## Introduction

Mast cells (MCs) are well known for their effector functions in T_H_2-skewed allergic inflammation, but have become increasingly acknowledged for their role in protection of health [1]. Human MCs are roughly divided into a binary phenotype depending on dominating protease content stored in granula, tryptase containing mast cells (MC_T_) and tryptase/chymase containing mast cells (MC_TC_), where the MC_T_ subtype dominates the airways in healthy conditions [2–4]. Since tryptase is a serine protease it can mediate effects via the G-protein receptor protease activated receptor 2 (PAR2) that is expressed on for example epithelial cells [5,6]. MC proteases have been shown to have both protective and harmful effects in respiratory tissue. MC proteases have a critical role in tissue homeostasis and wound repair, inducing and contributing with growth factors associated with tissue repair in pulmonary structural cells such as fibroblasts [7,8]. However, in pathological settings tryptase have shown in numerous studies not only to contribute to airway bronchoconstriction and hyperresponsiveness, but also to fibrosis and remodeling processes [9]. Positioned at environmental interfaces, such as the respiratory epithelium, MCs and their proteases can exert a rapid regulation of immune responses toward various stimuli [10]. Increases in the number of mast cells (MCs) in the lung are an important hallmark of asthma pathology [11,12] and elevated levels of tryptase have been observed in BAL fluid and serum from asthmatic patients and correlates with asthma severity [13,14]. However, the effect of MC tryptase on epithelial function remains incompletely understood.

Bronchial epithelial cells are vital for primary immune defense and lack of epithelial homeostasis is strongly associated with disease susceptibility and progression in chronic respiratory diseases such as asthma [15–17]. The respiratory epithelium constitutes a barrier from the external environment and has a key role in the innate immune system as a primary physical defense from allergen and/or pathogenic invasion [8,18]. It is established that in asthma, the airway epithelium is altered both in structure and function which manifest with increased mucus production, hyperplasia, damages and impaired viral and oxidant resistance [17,19]. In order to maintain a homeostatic barrier, an adequate and rapid epithelial wound repair is crucial to reestablish integrity and functions [20]. Epithelial repair is highly regulated and stimulates the intrinsic repair system which involves activation of epidermal growth factor receptor (EGFR) and ligands such as epidermal growth factor (EGF) and amphiregulin (AREG) which drives functional changes migration and proliferation [17,20,21]. Default or exaggerated repair response might contribute to an increase of profibrotic and growth factor release resulting in chronic airway remodeling [17,21].

The aim of this study is to investigate the normal physiological role and mechanism of MC tryptase on epithelial cell migration and proliferation in a wound gap model. To study this, we used a novel digital holographic cytometry (DHC) called Holomonitor M4 (Phase Holographic Imaging, Lund, Sweden). The advantage of this compared to traditional gap closure analyses is that it is a high throughput system, uses standard culture conditions with an automated image capture, segmentation and tracking of cells and combines functional aspects of cellular behavior with morphological properties on cell level. We hypothesize that tryptase will enhance gap closure in bronchial epithelial cells since earlier studies have established tryptase as a potent mitogenic for structural cells such as fibroblasts in the respiratory system [22,23].

## Material and methods

### Cell culture

Bronchial epithelial cell line BEAS-2B was purchased from ATCC (Walkersville, MD, USA). Cells were cultured in RPMI-medium 1640 (Gibco, 61,870–010) with addition of 10% fetal bovine serum (FBS) and 1% penicillin-streptomycin Life Technologies (Stockholm, Sweden). For harvest of supernatant, RNA and protein cells were cultured in six well nunc multidish (Nunc Technologies, Carlsbad USA). The cells were cultured at 37°C in 5% CO_2_ and were grown until confluency reached approximately 80–90%, automatically determined in the live cell imaging system Holomonitor Phase Holographic Imaging (Lund, Sweden) before start of the experiment. Thereafter, cells were treated with 0.5 μg/mL (equivalent to 31 mU/mL) human lung tryptase (Merck Millipore, Darmstadt, Germany). During stimulation of the cells starvation medium was used (RPMI medium with 1% FBS and 1% penicillin-streptomycin). Supernatants were collected after 1 hr, 6 hr and 24 hr post stimulation.

### RNA extraction and RT^2^ PCR array

Total RNA was harvested 6 hours post stimulation, isolated with RNeasy Plus Micro Kit from QIAGEN (Hilden, Germany) and measured with nanodrop 2000 c Thermo Scientific (Waltham Massachusetts USA). Thereafter RNA was transcribed into cDNA with RT^2^ First Strand Kit from QIAGEN (Hilden Germany) with a concentration of 1 μg in 111 μl RNase free water for PCR analysis (Agilent Technologies stratagene MX 3005P) using QIAGEN RT^2^ profiler PCR array for genes associated with wound healing pathways and inflammation. Targets in the profiling panel were chemokines, collagens, fibroblast agents, interleukins and matrix associated targets.

### Luminex and ELISA

For analyzing extra cellular protein secretion in cellular supernatant collected at 6 respective 24 hours. Luminex® MAGPIX® was used with commercially available kit Human XL Cytokine Discovery Fixed Panel from R&D systems (Minneapolis, USA). Regular sandwich ELISA, Human DuoSet from R&D systems (Minneapolis, USA) was also used for analysis of protein release.

### Immunocytochemistry

BEAS-2B were cultured in four well chamber slides (Merck Millipore, Darmstadt, Germany) until approximately 60% confluency. Cells were treated with tryptase for 24 h followed by fixation in 2% paraformaldehyde for 20 minutes and thereafter washed in PBS. Samples were heated in antigen retrieval buffer (EnVision DAKO, Glostrup, Denmark) using DAKO PT LINK (Glostrup, Denmark) version 2.0.0. Samples with intracellular targets were permeabilized in PBS containing 0.1% Tween-20 for 10 minutes. Samples were immunostained with antibodies against KI67 (1:300) and CD151 (dilution 1:150) DAKO, Glostrup, Denmark and Sigma-Aldrich, St Louis, MO, USA, respectively) according to standard protocols. Antibodies against KI67 and CD151 were labeled with Alexa Fluor 488 diluted 1:200 (Invitrogen, Eugene, USA). Nuclei were counterstained with mounting medium ProLong Gold antifade reagent with DAPI (Invitrogen, Eugene, USA). Samples were viewed on Nikon eclipse 80i (Melville, NY, USA) combined with Nikon DS-QI1MC. Color intensity was quantified using ImageJ (Wisconsin, USA) by measuring the pixel intensity in relation to the threshold value which was determined based on untreated controls. The intensity measurement in these randomly chosen areas was then divided by the number of cells (based on nuclei DAPI staining) in each field of view.

### Scanning electron microscopy (SEM)

BEAS-2B were fixed in approximately ten times the sample volume of fixation solution containing 0.1 M Sorensen´s phosphate buffer pH 7.4, 1.5% formaldehyde and 1.5% glutaraldehyde at room temperature for 20 min. After fixation the samples were washed twice in 0.1 M Sorensen´s buffer pH 7.4 to remove excess fixative. Samples were then dehydrated in a graded series of ethanol (50%, 70%, 80%, 90% and twice in 100%) and subsequently critical point dried before being mounted and examined in a Jeol JSM-7800 F FEG-SEM.

### Live cell imaging: gap closure and migration assay

For live cell imaging analysis for epithelial gap closure, migration and morphology, BEAS-2B were cultured in Sarstedt TC 6-well plate (Nümbrecht Germany). Scratch was created with HoloLid 71,120 (Lund, Sweden) culture-insert 2 well (Ibidi, Martinsried, Germany) where 70 μL cell suspension (5*10^5^ cells/mL) was applied into each well and cultured for 24 hours to reach confluency, thereafter the inserts were removed. Cells were treated with tryptase 0.5 μg/mL for 24 hours and monitored in live cell imaging system Holomonitor Phase Holographic Imaging (Lund, Sweden). For assay with PAR2 inhibition BEAS-2B were pre-treated for 30 minutes in 37°C in 5% CO_2_ with antagonist I-191 HY117793 (Sollentuna, Sweden). After incubation with I-191 BEAS-2B were washed in PBS and thereafter 0.5 μg/mL tryptase or starvation RPMI-medium only was added to the wells. Functional studies of epithelial gap closure, migration and morphology were performed using HoloMonitor M4 live cell imaging system Phase Holographic Imaging (Lund, Sweden). In the present study following parameters was investigated in order to evaluate the wound gap closure: gap width (micrometers), cell covered area (%, statistically quantified as fold change), motility (micrometers), motility speed (micrometer/hour) and morphology (boxed length). A capture pattern for each well was selected, in this project three randomly chosen positions in each well were selected. Time-lapse was selected to 24 hours monitoring with images taken every 7 minutes of the three selected positions in each well. The monitoring of one six-well plate resulted in approximately 3700 images. Quantitative phase imaging of wound healing followed by automatic cell tracking to determine migration was performed using automated acquisition and image segmentation of multiple time-lapse image sequences in multiple wells in HStudio [24]. A minimum of 9 randomly chosen cells per technical repeat (in total minimum 27 cells) were analyzed per stimulation. Regarding the use of the term migration, we will refer to two quantitative parameters: migration (motility) and migration (x-axis). Migration (motility) refers to the quantification of the random cellular movement with the total sum of covered distance measured in micrometers over the duration of imaging. This parameter does not measure directness of the movement toward a certain target. To investigate the cell movement into the gap we used a parameter called ‘migration (x-axis)’. This is the quantification of cell movement in micrometers of cells migrating into the gap on an x-axis position since all the gaps were created in a vertical way. For quantification of cell morphology, the parameter boxed length (µm) was used. Briefly, a frame is fitted around the cell and the frame that fits the cell while covering the smallest area is selected for measurements. The boxed length is the inside length of the rectangle that precisely encloses the whole cell.

### Statistical analysis

Statistical analysis was performed using GraphPad Prism 7.0 (GraphPad Software, La Jolla, CA). Nonparametric test including Mann-Whitney U test were used to detect differences between 2 groups and Kruskal Wallis test with Bonferroni post hoc test to detect differences between more than 2 groups. Results were considered significant at p ≤ 0.05.

## Results

### Mast cell tryptase enhances bronchial epithelial wound gap closure

**Figure 1.**
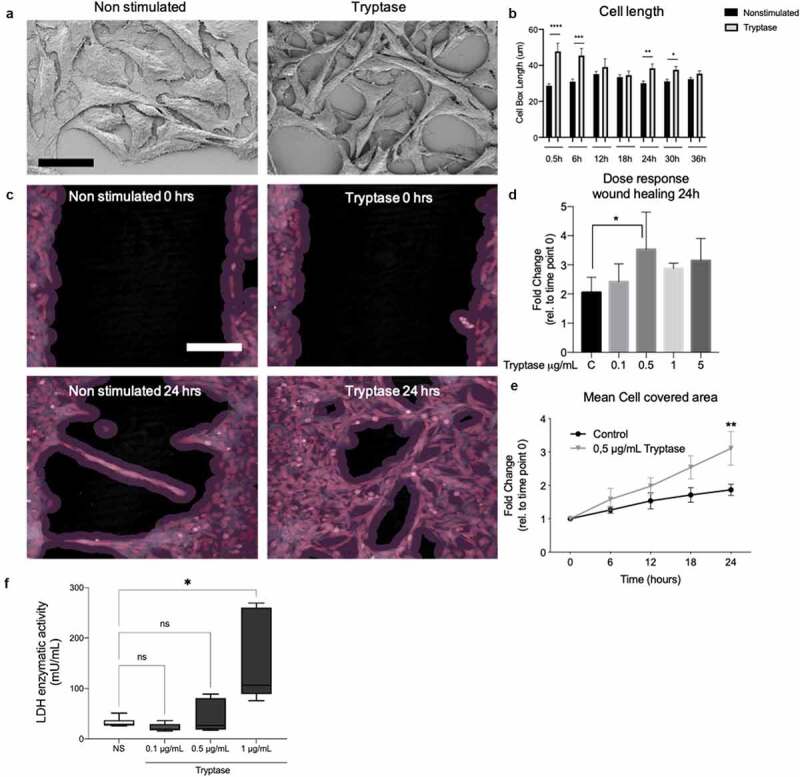
MC tryptase increased cell length and enhanced epithelial gap closure in a wound gap model. Altered cell morphology was observed using scanning electron microscopy (SEM) in non-stimulated and tryptase stimulated BEAS-2B (a). Increased cell box length was seen using live cell imaging in tryptase stimulated cells compared to non-stimulated cells (b). Representative holographic images of gap closure in BEAS-2B in non-stimulated and tryptase stimulated cells (c). Dose-response of tryptase (0.1, 0.5, 1.0 5.0 μg/mL) on epithelial gap closure in BEAS-2B (d). Comparison of gap closure between non-stimulated and tryptase stimulated BEAS-2B, expressed as fold change of mean cell covered area (%) (e). Lactate dehydrogenase (LDH) cytotoxicity test of tryptase for epithelial cells for estimating tryptase working concentration (f). Statistical significance between non-stimulated and tryptase stimulated cells was tested using Mann-Whitney or Kruskal-Wallis (D) . *P < 0.05, **P < 0.01, ***P < 0.001 and ****P < 0.0001. Scale bar in C: 200 µm. Results based on three monitored positions in each well in three technical repeats per stimulation

Using SEM, BEAS-2B treated with 0.5 μg/mL tryptase had an elongated morphology compared to non-stimulated cells (Figure 1(a-b)). This observation was confirmed in ananalysis of cell boxed length measured in µm in a wound gap model using live cell imaging system from Phase Holographic. We observed differences of cell length between non-stimulated and tryptase stimulated cells at both early and late timepoints during the 36 hour live cell monitoring. Tryptase treated cells was significantly elongated at 30 minutes (47.80 µm ± 4.481, p < 0.0001) compared to non-stimulated cells (28.66 µm ±1.187). Tryptase-stimulated cells remained significantly elongated at 6 hrs, 24 hrs and 30 hrs compared to non-stimulated controls (6h: 45.47 µm ±3.971, p = 0.0003; 24 h: 38.40 µm ±2.426, p = 0.007; 30 h: 37.60 µm ±1.863, p = 0.02) (Figure 1(b)). The possible mitogenic effect of tryptase was further explored in the wound gap model. Prior to starting a dose response analysis was performed and we found that tryptase enhanced epithelial gap closure (cell covered area %) at plural concentrations but was significant at the dose 0.5 μg/mL (p = 0.013) (Figure 1(d)). Monitoring the BEAS-2B for 24 hours we identified a trend for enhanced gap closuring effect of tryptase stimulated cells compared to untreated controls at several timepoint, however at 24 h tryptase stimulated cells had significant improved gap closure compered to untreated controls (non-stimulated:1.475 fold change±0.1553, tryptase 2.040 fold change±0.3661, p = 0.003). (Figure 1(c), 1(e)).

### Mast cell tryptase enhances wound gap closure by inducing increased cellular migration and speed in bronchial epithelial cells

**Figure 2.**
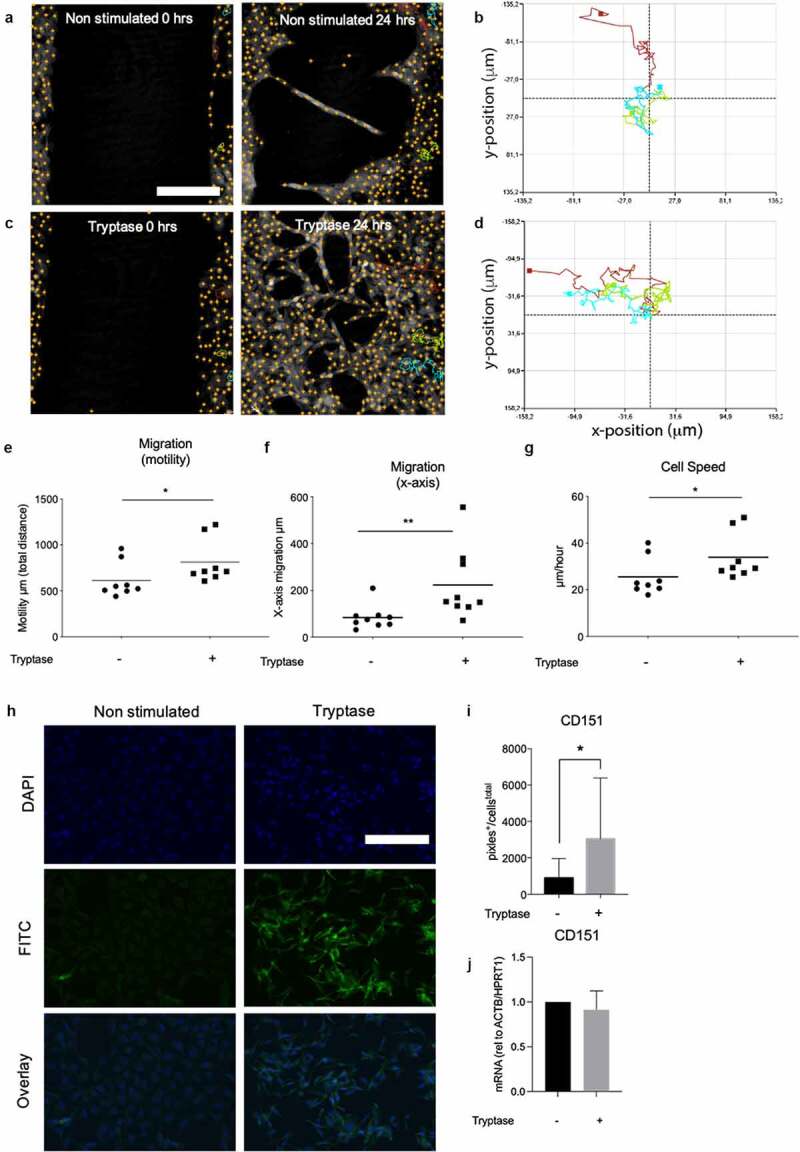
MC tryptase induced increased cellular migration, motility and cell speed in a wound gap model in BEAS-2B. Representative images of single cell tracking from a representative position in non-stimulated cells (a) and tryptase stimulated cells (c). Migration plots of total distance and direction in three non-stimulated cells (b) and three tryptase stimulated cells (d) from a representative focus point in one technical repeat. Tryptase stimulated BEAS-2B showed increased total migrated distance (μm, motility) (e), migration in x-axis (μm) (f) and average speed of cell populations (μm/hour) (g) in comparison to non-stimulated cells. Representative micrographs of immunofluorescence stain for CD151 (h). Quantification of CD151 in BEAS-2B as protein expression (pixels^+^/cells^total^) (i) and mRNA expression (relative to ACTB/HPRT1) (j) with and without tryptase stimulation. Statistical significance between non-stimulated and tryptase stimulated cells was tested using Mann-Whitney. *P < 0.05 and **P < 0.01. Scale bar in A: 250 µm and H: 70 µm. *Migration*: results based on randomly chosen individual cell tracking of three monitored positions in each well in three technical repeats (in total a minimum of 27 tracked cells per stimulation). *Immunofluorescence*: Results based on analysis of all cells in minimum of three randomly chosen fields of view (20x magnification) in three technical repeats per stimulation

The use of holographic live cell imaging enables studies of parameters contributing to the enhanced gap closure in the same experimental set up. We further studied the cellular migration in the wound gap closure assay that was monitored using live cell imaging system from Phase Holographic. Post live cell imaging, multiple individual cells in the wound edges were identified and single cell tracked throughout the 24-hour capturing (Figure 2(a, c)). The holographic software system provides migration pattern and direction of the single cell tracking displayed as a plot chart (Figure 2(b, d)). We found that tryptase stimulated cells had an improved total distance migration (motility) 813 µm ±84.91 (p = 0.04) and migration directness defined by cells migrating toward the gap (x-axis) 223.4 µm ± 50.62(p = 0.005) compared to non-stimulated cells (motility: 614.3 µm ±67.72 and x-axis 83.80 µm ±17.03) (Figure 2(e-f)). We also found that the cellular migration speed was also upregulated by tryptase stimulation (33.92 µm /h ± 3.534 p = 0.05) compared to non-stimulated cells (25.52 µm /h ± 2.877) in BEAS-2B (Figure 2(g)). Immunofluorescence staining of the migration marker CD151 displayed on protein level that tryptase stimulated cells had increased expression of CD151 (3036 pixels^+^/cells^total^±1119 p = 0.05) compared to non-stimulated cells (880.5 pixels^+^/cells^total^ ±367.3). (Figure 2(h-i)).

### Tryptase induces proliferation on both gene and protein level in bronchial epithelial cells

**Figure 3.**
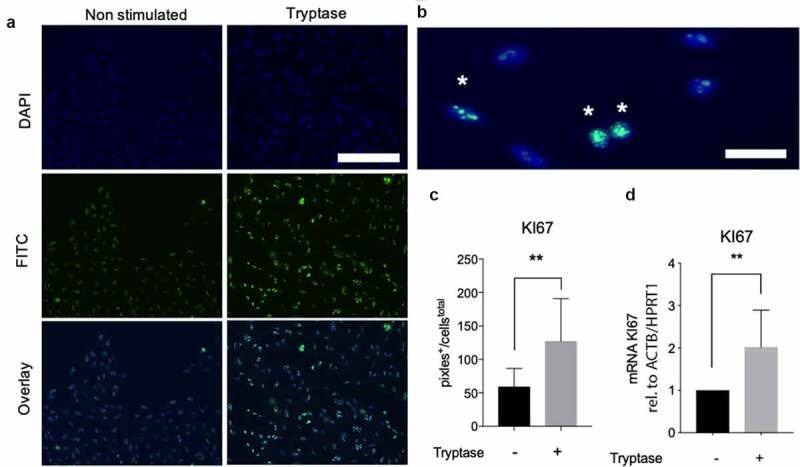
MC tryptase increased expression of KI67 on both protein and gene level. Representative micrographs of immunofluorescence stain for KI67 (FITC, green) and nuclei (DAPI, blue) (a). High magnification image of bronchial epithelial cells where * denotes KI67 positive cells (b). Image shows clear intranuclear KI67 staining that confirms validity of the staining method and antibody. Quantification of immunofluorescence stain for KI67 expression (pixels^+^/cells^total^) in BEAS-2B (c) and mRNA expression (relative to ACTB/HPRT1) of KI67 in BEAS-2B with and without tryptase stimulation (d). Statistical significance between non-stimulated and tryptase stimulated cells was tested using Mann-Whitney. *P < 0.05 and **P < 0.01. Scale bar in A: 70 µm and B: 40 µm. *Immunofluorescence*: Results based on analysis of all cells in minimum of three randomly chosen fields of view (20x magnification) in three technical repeats per stimulation. *mRNA*: Results based on a minimum of four technical repeats per stimulation

Detection of the proliferation marker KI67 with immunofluorescence staining and quantification of pixel intensity (Figure 3(a-b)) revealed that tryptase upregulated proliferation (127.1 pixels^+^/cells^total^ ± 19.26 p = 0.004) on protein level compared to untreated cells (58.96 pixels^+^/cells^total^ ±8.322) in BEAS-2B (Figure 3(c)). Higher mRNA levels of KI67 were also found in tryptase stimulated cells (2.023 foldchange ± 0.3879 p = 0.007) compared to non-stimulated cells (Figure 3(d)).

### Tryptase upregulates expression of growth factors associated with asthma and wound healing pathways in bronchial epithelial cells

**Figure 4.**
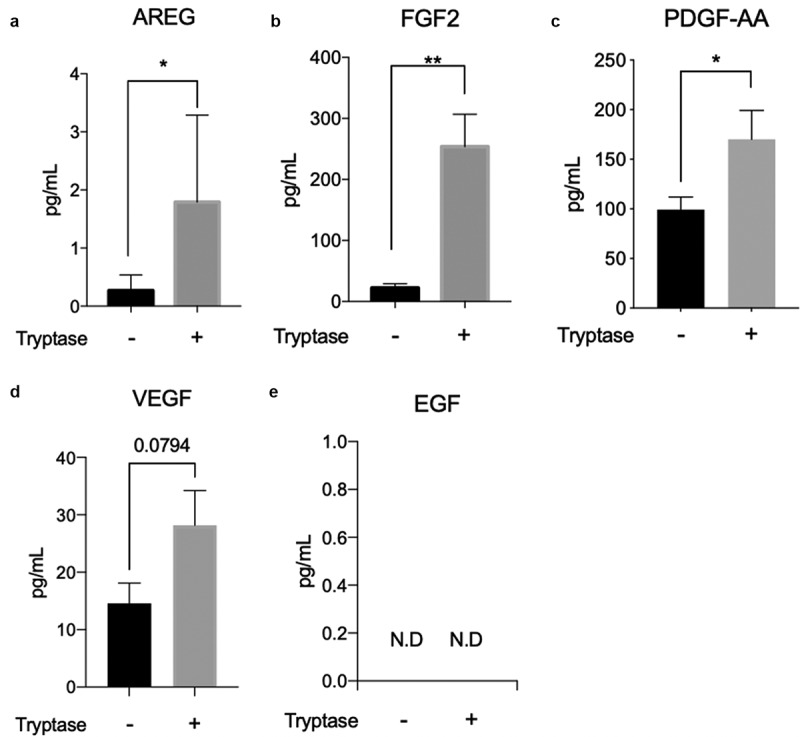
Tryptase induced release of growth factors AREG, FGF2, PGDF-AA in BEAS-2B. Comparison of growth factor release between non-stimulated and tryptase stimulated cells using ELISA and Luminex (a-e). Levels of amphiregulin (AREG) (a), fibroblast growth factor 2 (FGF2) (b), platelet derived growth factor-AA (PDGF-AA) (c), vascular endothelial growth factor (VEGF) (d) and epidermal growth factor (EGF) (e) in culture supernatant (pg/mL). Statistical significance between non-stimulated and tryptase stimulated cells was tested using Mann-Whitney. *P < 0.05 and **P < 0.01. N.D (not detectable). Results based on a minimum of four technical repeats per stimulation

Comparison between tryptase stimulated cells and non-stimulated cells using RT^2^ PCR profiler array for asthma and allergy and wound healing pathways showed that tryptase alone upregulated AREG (fold difference 6.35, p = 0.004), IL12B (fold difference 4.00, p = 0.02), CCL2 (fold difference 4.00, p = 0.03), CHI3L1 (fold difference 3.17, p = 0.03) and CCR4 (fold difference 2.52, p = 0.02) on gene level. FGF2 and VEGF was upregulated but did not reach significance (Table 1). Using ELISA and Luminex® we further investigated the growth factors on protein level. In tryptase stimulated cells there was a significant increased protein release of AREG (1.792 pg/ml±0.6698, p = 0.03), FGF2 (253.7 pg/ml±23.77, p = 0.008) and PDGF-AA (170.0 pg/ml± 14.67, p = 0.03) (Figure 4(a-c)) compared to non-stimulated cells (AREG: 0.2693 pg/ml±0.1199; FGF2: 22.16 pg/ml± 3.137 and PDGF-AA: 99.33 ± 6.375). The release of VEGF was not significant upregulated in tryptase treated cells (Figure 4(d)) and EGF was not detectable on protein level in BEAS-2B (Figure 4(e)).

**Table 1.** Up- and downregulated genes related to wound healing in bronchial epithelial cells post tryptase stimulation

Gene Symbol	Gene name	Fold difference	P value
AREG	Amphiregulin	6.35	0.0039
IL12B	Interleukin 12B	4.00	0.024
CCL2	Chemokine (C-C motif) ligand 2	4.00	0.028
CHI3L1	Chitinase-3-like protein 1	3.17	0.027
CCR4	Chemokine (C-C motif) receptor 4	2.52	0.021
CDH1	Cadherin 1, type 1, E-cadherin	*−1.59*	*ns*
CTGF	Connective tissue growth factor	*2.16*	*ns*
CTNNB1	Catenin (cadherin-associated protein)	*1.59*	*ns*
EGF	Epidermal growth factor	*1.0*	*ns*
EGFR	Epidermal growth factor receptor	*−1.59*	*ns*
FGF2	Fibroblast growth factor 2	*1.36*	*ns*
VEGF	Vascular endothelial growth factor A	*1.36*	*ns*
PDGFA	Platelet-derived growth factor alpha	*−1.26*	*ns*

Fold regulation comparison and p-value using RT2 profiler array for wound healing in three technical repeats. A fold change over/under 2 with a p-value <0.05 was considered significant. ns: non-significant.

### PAR2 antagonist I-191 impair tryptase induced gap closure, cellular migration and release of growth factor in human bronchial epithelial cells

**Figure 5.**
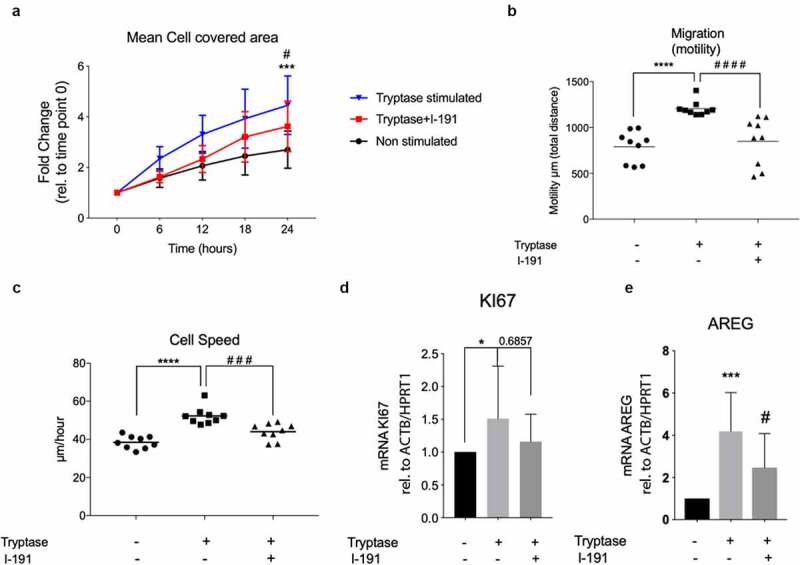
PAR2 inhibitor I-191 decreased wound gap closure, migration, proliferation and cell speed in tryptase stimulated BEAS-2B. Comparison of gap closure in BEAS-2B between non-stimulated, tryptase stimulated and tryptase stimulated cells with I-191 pre-treatment, expressed as fold change of mean cell covered area relative to the starting point (a). Comparison of migration (μm, motility) (b) and cell speed (μm/hour) (c) between non-stimulated, tryptase stimulated and tryptase stimulated cells with I-191 pre-treatment. mRNA expression of proliferation marker KI67 (d) and growth factor amphiregulin (AREG) (e) in non-stimulated, tryptase stimulated and tryptase stimulated cells with I-191 pre-treatment (relative to ACTB/HPRT1). Results were considered significant at p ≤ 0.05, and individual differences between groups using Kruskal–Wallis test with Dunn’s *post hoc* test. ***P < 0.001 and ****P < 0.0001 denotes comparison between non-stimulated and tryptase stimulated cells, *^#^P* < 0.05 *^##^P* < 0.0501and ^####^*P* < 0.0001 denotes comparison between tryptase stimulated cells and cells stimulated with I-191+ tryptase. *Migration*: results based on randomly chosen individual cell tracking of three monitored positions in each well in three technical repeats (in total a minimum of 27 tracked cells per stimulation). *mRNA*: Results based on a minimum of four technical repeats per stimulation

To further investigate a potential mechanism behind the pro-migratory effect of tryptase we treated BEAS-2B with the selective PAR2 antagonist I-191. Using a wound gap model in holographic live cell imaging we observed that tryptase stimulated BEAS-2B pre-treated with PAR2 inhibitor I-191 had a decreased capacity of epithelial gap closure (2.358 fold change ±0.4853) compared to cells stimulated with tryptase only (3.006 fold change±0.6128, p = 0.04) (Figure 5(a)). Investigation of cellular migration in the presence of the PAR2 antagonist showed that tryptase induced migration (motility) and migration speed was downregulated by PAR2 treatment to a similar level as untreated controls. (Figure 5(b-c)). Tryptase treated cells in the absence of PAR2 inhibitor yet manifested an improved motility in total migrated µm (1260 µm ± 27.21, p < 0.0001) compared to non-stimulated cells (790 µm ± 57.27). However, cells treated with I-191 prior addition of tryptase displayed an impaired capacity of motility in terms of total migrated distance in µm (849.1 µm ±87.08, p < 0.0001) compared to cells treated with tryptase only (1260 µm ± 27.21 (Figure 5(b)). The same pattern was observed for the cellular migration speed (µm/h) where cells treated with I-191 prior tryptase stimulation exhibited a reduced migration speed (44.06 µm /h ± 1.449) compared to cells treated with tryptase only (52.30 µm /h ± 1.527, p = 0.0003). We also measured mRNA levels of proliferation marker KI67 after pre-treatment with I-191, although it was confirmed again that tryptase increase KI67 compared to non-stimulated cells (p = 0.03) no significant effect on KI67 in tryptase stimulated cells pre-treated with I-191 was found (Figure 5(d)). However, tryptase stimulated cells, pre-treated with PAR2 antagonist I-191 had a reduced gene expression of growth factor AREG compared to cells treated with tryptase only (p = 0.05) (Figure 5(d)).

## Discussion

In summary our data showed that the MC mediator tryptase enhances epithelial wound gap closure by increasing migration proliferation and secretion of growth factors in normal BEAS-2B, which was confirmed at gene level, protein and functional level. Our results also suggest that tryptase activation of PAR2 is responsible for the enhanced epithelial migration and closing of wound gaps in bronchial epithelial cells and may in part be dependent on tryptase induced epithelial production of AREG. MC derived tryptase might thus have an important role in maintaining epithelial barrier function.

The overall result of this project confirms earlier studies that tryptase acts as potent mitogenic [23,25,26]. However, current knowledge of tryptase epithelial interactions is pre-dominantly based on cells of non-pulmonary origin [27]. Since MCs are strongly associated with asthma and MC release occurs in pulmonary microenvironments, a closer investigation of tryptase and epithelial interactions is warranted for a detailed understanding of tryptase contribution in asthma pathology and the possibility of using tryptase as a clinical tool for disease progression or prognosis [27]. In 1996, it was described that tryptase promotes DNA synthesis in epithelial cells [23]. We confirm these findings and further advance this by an observed enhanced capacity of proliferation revealed by the increase of the nuclei target KI67. Furthermore, our results also suggest that tryptase activation of PAR2 is responsible for the enhanced epithelial migration and closing of wound gaps in bronchial epithelial cells. Although the effect of MC proteases has been studied in other epithelial cell types and structural cells [23,25,28–30], less is known regarding the effect on epithelial cells of lung origin. We have recently shown that MC tryptase and chymase have a strong effect on bronchial epithelial proliferation and morphology using live cell imaging [31]. Little is however known regarding the tryptase effect on bronchial epithelial wound healing processes and the involvement of PAR2 signaling using live cell imaging.

Epithelial proliferation is required to close wound gaps and it is mediated by a wide range of growth factors and cytokines [32,33]. The proliferative response in the airway epithelium may be triggered by viral infection or injury in susceptible individuals. Excessive growth and proliferation of epithelial cells might also have detrimental effects. Cohen *et al*. found increasing numbers of proliferating bronchial epithelial cells positive for the nuclear antigen KI67 that increased with asthma severity [34]. KI67 have also been described to increase in COPD current smoker compared to ex-smokers. Here, the number of KI67 positive epithelial cells correlated positively to squamous cell metaplasia and expression of EGFR within the epithelium [35]. Interestingly, both severity of asthma and COPD are associated with increased number of MCs [2,36,37]. Increased epithelial proliferation may manifest as epithelial hyperplasia and thickening resulting in airway remodeling and result in persistent physiologic abnormalities. In the present study tryptase improves proliferation in bronchial epithelial cells, which was revealed as upregulation of KI67 on both protein and gene level (Figure 3(a-d)) [23]. The proliferative effect of tryptase has earlier been described in fibroblasts and studies have suggested that tryptase induce the proliferation in fibroblasts via PAR-2 [22,38]. In airway smooth muscle (ASM) cells stimulated with tryptase no effect on proliferation was noted when tryptase’s catalytic activity was inhibited [39]. Our proliferation data on BEAS-2B demonstrates similar results: blocking of PAR2 in our experimental system causes no complete blocking of the proliferative change seen in tryptase stimulated cells (Figure 5(d)). Although a trend toward a decrease was observed, this might indicate that tryptase have PAR2 independent proliferative effects in epithelial cells.

Whether tryptase enhanced wound gap closure could be caused by changes in epithelial cell migration was furthermore investigated in the present study. A rapid epithelial repair post injury caused by pathogens, allergens or pollutants is crucial to maintain homeostasis. Epithelial migration is one of the primary responses upon injury and an important factor for the gap closing process where a temporary barrier is arranged [17,20,40,41]. To our knowledge, the results obtained in the present study demonstrated for the first time that MC tryptase alone is a potent inducer of PAR2 dependent migration in human bronchial epithelial cells (Figure 5(b)). The present study has shown that MC tryptase enhanced wound healing in human bronchial epithelial cells (Figure 1(e)) by increasing both random movement (Figure 2(e)) and directed cellular migration into the gap (figure 2(f)), as well as increasing migration speed (Figure 2(g)). It has earlier been described that tryptase enhance gap closure in fibroblast wound healing models and it is well recognized that MC tryptase stimulates fibroblast and retinal pigment epithelial migration [22,25,26]. Cell migration is a complex process with rearrangement of cytoskeletal proteins and rearrangement of cell surface proteins. CD151 is a member of the tetraspanin family and the encoded protein is a cell surface glycoprotein that is known to form complex with for example integrins. It is involved in cellular processes including cell adhesion, enhanced cell motility and may regulate integrin trafficking and/or function [42–44]. Our study showed that tryptase upregulates CD151 (Figure 2(i)) and can thereby enhance epithelial cellular migration. Our findings also correlates with earlier results from a murine model where CD151 null mice displayed unstable hemostasis and defective migration ability in a wound healing model [44].

Other factors that cause migratory effects in epithelial cells are growth factors TGF-β, EGF and AREG which have been shown to increase in asthma [45,46]. Our study has shown that tryptase promotes expression of growth factors FGF2 and PDGF-AA in bronchial epithelial cells (Figure 4(b-c)). Furthermore, we showed that tryptase upregulated AREG (Figure 4(a)), which earlier been shown to induce epithelial as well as fibroblast proliferation. However, it remains unknown whether this contributes to needed tissue repair or if it can, in pathology lead to fibrosis and airway remodeling [45,47]. It should be noted that there was a lack of conformity between the mRNA and protein data for some of the growth factors (as well as CD151). This might be explained by the possible use of the wrong time-point or that some mediators are prestored with in the epithelial cell and can thus be released very quickly upon stimulation.

Tryptase is a serine-class protease of the trypsin family and appears in a diversity of form, activity and pattern expression. The soluble ß-tryptases are the main form of tryptase stored in MCs and are packed in high concentration within the granule [9,48,49]. In our study tryptase derived from human lung was used and should thus have comparable composition as what is stored in MC granules *in vivo*. In our experiments the lowest possible dose of tryptase that had an effect on gap closure was used. This concentration is within the range (at the lower limit) of what has been used in other experimental set ups with respiratory structural cells [23,50]. No toxic effects of tryptase (sloughing or increased LDH levels) on epithelial cells with any of the concentrations used in the dose-response analysis were seen. By using a relatively low dose of tryptase, we aimed to mimic the effect of MC tryptase under healthy baseline condition with the intention to investigate the role of MC tryptase in epithelial homeostasis.

We have in the present paper showed that low levels of MC tryptase have important effects on basic epithelial cell functions such as proliferation and migration, all important for an effective healing of damages to the epithelial layer. The use of BEAS-2B cells enables us to investigate these effects in a normal, healthy environment. As MCs are rare within or near the epithelium in normal conditions, and since they are not activated by anaphylaxis (but rather piecemeal degranulation), the levels of tryptase are likely to be low under normal circumstances. This indicates an important role for MCs and tryptase in maintaining epithelial homeostasis. However, future research should focus on investigating the effect of different concentrations of tryptase in pathological conditions that are characterized by increased number of MCs such as asthma. Apart from a heightened inflammatory response with recruitment of immune cells, elevated levels of tryptase are likely to cause a further increased secretion of growth factors and proliferation in epithelial cells which could lead to hyperplasia and remodeling, and subsequently, loss of a functional barrier.

It is difficult to determine how much tryptase is released *in vivo* as well as the local concentration around each epithelial cell when MCs degranulate. Due to that MC proteases are secreted in large aggregates, complexed with serglycin proteoglycans, and these complexes tend to accumulate locally [27], the local concentration of tryptase around individual cells is likely to be quite high and wound healing would likely be affected in a negative way. Increased levels of tryptase has been correlated to disease severity in asthmatic patients [13]. Tryptase positive MC have also been observed in bronchial histological samples from severe asthmatic patients with an airway epithelium characterized as hyperproliferative and metaplastic which could indicate a possible relation between MCs and epithelial remodeling and loss of function [51]. However, MCs also secrete mediators through piecemeal degranulation, secreting lower amounts of tryptase [52], where the effects on the airway epithelial cell might be more wide-ranging. Hence, it would be of great interest to investigate this further in primary epithelial cells from different phenotypes of asthmatic patients would increase our knowledge on the possible detrimental or positive effects of tryptase in these settings. There is great interest in tryptase as a therapeutic target mainly for the treatment of inflammatory diseases such as asthma and inflammatory bowel disease [53,54]. Since there is a vast body of evidence that tryptase is involved in structural alterations of the airway mucosa, inhibitors of tryptase would meet the demand for new therapies of many chronic airway diseases, since steroids have shown few beneficial effects on airway remodeling. However, this is an area that warrants further investigation since tryptase most likely both have beneficial and detrimental roles on epithelial homeostasis.

In conclusion, this study showed that tryptase has a potent mitogenic effect on both migration and proliferation in normal bronchial epithelial cells BEAS-2B as well as increased epithelial production of several growth factors. We can also report that the pro-migratory, but not proliferative, effect of tryptase is PAR2 dependent. This suggest a pivotal role of MC tryptase in healthy tissues in terms of rapid gap closure in the damaged epithelial barrier. However, in a pathological context tryptase could be harmful, having a catalytic role in chronic inflammation and remodeling as well as enhancing tumor progression via pro-migratory effects. Our results highlight that further research is warranted given the possibility to target tryptase and PAR2 for clinical therapeutic use.
